# Reduced mitophagy is an early feature of NAFLD and liver-specific PARKIN knockout hastens the onset of steatosis, inflammation and fibrosis

**DOI:** 10.1038/s41598-023-34710-x

**Published:** 2023-05-10

**Authors:** R. Undamatla, O. G. Fagunloye, J. Chen, L. R. Edmunds, A. Murali, A. Mills, B. Xie, M. M. Pangburn, I. Sipula, G. Gibson, C. St. Croix, M. J. Jurczak

**Affiliations:** 1 Division of Endocrinology and Metabolism, Department of Medicine, School of Medicine, University of Pittsburgh, 200 Lothrop Street, BST W1060, Pittsburgh, PA 15213 USA; 2 Department of Cell Biology, Center for Biologic Imaging, University of Pittsburgh, Pittsburgh, PA USA; 3 Center for Metabolism and Mitochondrial Medicine, University of Pittsburgh School of Medicine, Pittsburgh, PA USA

**Keywords:** Non-alcoholic fatty liver disease, Mitochondria

## Abstract

Nonalcoholic fatty liver disease (NAFLD) encompasses a spectrum of pathologies that includes steatosis, steatohepatitis (NASH) and fibrosis and is strongly associated with insulin resistance and type 2 diabetes. Changes in mitochondrial function are implicated in the pathogenesis of NAFLD, particularly in the transition from steatosis to NASH. Mitophagy is a mitochondrial quality control mechanism that allows for the selective removal of damaged mitochondria from the cell via the autophagy pathway. While past work demonstrated a negative association between liver fat content and rates of mitophagy, when changes in mitophagy occur during the pathogenesis of NAFLD and whether such changes contribute to the primary endpoints associated with the disease are currently poorly defined. We therefore undertook the studies described here to establish when alterations in mitophagy occur during the pathogenesis of NAFLD, as well as to determine the effects of genetic inhibition of mitophagy via conditional deletion of a key mitophagy regulator, PARKIN, on the development of steatosis, insulin resistance, inflammation and fibrosis. We find that loss of mitophagy occurs early in the pathogenesis of NAFLD and that loss of PARKIN accelerates the onset of key NAFLD disease features. These observations suggest that loss of mitochondrial quality control in response to nutritional stress may contribute to mitochondrial dysfunction and the pathogenesis of NAFLD.

## Introduction

Nonalcoholic fatty liver disease (NAFLD) is a global health epidemic that is characterized by excessive liver fat accumulation, otherwise known as steatosis, in hepatocytes in the absence of significant alcohol intake or other secondary causes of hepatic steatosis. This chronic disease encompasses a broad clinical spectrum ranging from hepatic steatosis (also known as NAFL, fat accumulation in more than 5% of hepatocytes), nonalcoholic steatohepatitis (NASH, characterized by the presence of hepatocellular damage, ballooning, inflammation with or without fibrosis), advanced fibrosis (cirrhosis), and hepatocellular carcinoma^1,2^. Although NAFL is considered benign and more readily reversible, without proper intervention, it may progress to NASH and advance to end-stage liver disease^3^. In addition, NAFLD is closely associated with hallmarks of metabolic syndrome, including obesity, insulin resistance, hyperglycemia, type 2 diabetes, and dyslipidemia^4^, such that a new name for the disorder, metabolic associated fatty liver disease or MAFLD, was recently proposed^5^. While knowledge about the underlying causes of NAFLD is still evolving, genetic factors and epigenetic factors, as well as environmental factors such as consumption of a western diet, loss of physical activity or increased body weight, are all believed to contribute to NAFLD pathogenesis^6^.

Changes in mitochondrial function are also implicated in the pathogenesis of NAFLD and may contribute to what is often referred to as the ‘second hit’ required to transition from NAFL to NASH^7^. Although data from human studies is somewhat limited due to challenges in collecting liver samples, particularly from healthy control subjects, a growing number of studies support the hypothesis that changes in mitochondrial function are associated with NAFLD. For example, hepatic ATP turnover, a surrogate for mitochondrial function, was reduced in patients with type 2 diabetes or NASH^8,9^. Hepatic mitochondrial structural defects and increased oxidative stress were positively associated with insulin resistance and steatosis in patients with NAFLD and NASH^10,11^. Moreover, a first of its kind study where liver biopsies were collected from healthy patients, obese patients with and without simple hepatic steatosis, and obese patients with NASH demonstrated increased mitochondrial respiratory capacity that occurred in response to obesity that was subsequently lost after the onset of NASH^12^. Interestingly, liver mitochondrial mass was not different between lean and obese subjects, but was increased in patients with NASH, despite reduced respiratory capacity^12^. This specific observation, namely increased mitochondrial content with impaired function, suggested loss of the mitochondrial quality control pathway known as mitophagy that selectively degrades damaged mitochondria via the autophagosomal/lysosomal pathway^13^. The idea that mitophagy flux is paradoxically reduced in the setting of hepatic steatosis and mitochondrial dysfunction was directly assessed using the mitophagy reporter mouse, mito-Keima, where 18 weeks of high-fat diet feeding significantly reduced relative rates of mitophagy in liver^14^. These observations raise the intriguing possibility that defective hepatic mitophagy enhances obesity-associated liver metabolic disease and contributes to the pathogenesis of NAFLD.

To test this idea directly, we recently characterized a liver-specific *Prkn* knockout model (LKO)^15^. *Prkn* encodes an E3 ubiquitin ligase, PARKIN, that plays a key role in mitophagy signaling. PARKIN-mediated mitophagic signaling occurs specifically on the outer membrane of damaged mitochondria and requires the coordinated recruitment and activation of PTEN-induced putative kinase-1 (PINK1) and PARKIN^16^. Full recruitment and activation of PARKIN is dependent on phosphorylation of both PARKIN and outer mitochondrial membrane ubiquitin by PINK1, resulting in ubiquitination of outer mitochondrial membrane proteins by PARKIN, marking the damaged mitochondria for autophagosomal degradation^17–24^. We found that reduced mitophagy was not simply an associative feature of fatty liver but that the loss of PARKIN-mediated mitophagy impaired mitochondrial respriratory capacity and exacerbated both liver fat accumulation and the severity of insulin resistance after high-fat diet feeding^15^. Notably, the previous observations made in the mito-Keima mice and our own work with the liver-specific PARKIN knockout mice utilized high-fat diets that modeled the metabolic aspects of NAFLD (steatosis and insulin resistance) but not the inflammatory or fibrotic features^25,26^, and both were limited to single dietary durations (18 and 12 weeks, respectively for mito-Keima and LKO studies). In order to determine when relative rates of mitophagy decline during the pathogenesis of NAFLD, as well as how the loss of PARKIN-mitophagy contributes to the early and late features of NAFLD, we performed time course studies in mito-Keima and LKO mice using a well-established dietary model that induces the full spectrum of NAFLD pathology, specifically steatosis, inflammation and fibrosis^25^.

## Materials and methods

### Animal care and use

Mice were bred and housed at the University of Pittsburgh. All studies were reviewed and approved by the Institutional Animal Care and Use Committee at the University of Pittsburgh prior to initiation and are consistent with ARRIVE guidelines. All experiments were performed in accordance with the relevant guidelines and regulations. Liver-specific PARKIN knockout mice (LKO) on the C57BL6/J background were generated, as previously described^15^, by crossing *Prkn* floxed mice (*Prkn*^fl/fl^) with Albumin-Cre (*Albcre*) mice sourced from Jackson Labs (stock #003574). Male and female littermate control wild-type (*Prkn*^fl/fl^-*Albcre*^*−*/*−*^ or WT) and experimental LKO (*Prkn*^fl/fl^-*Albcre*^*−*/+^) mice were generated by breeding *Prkn*^fl/fl^-*Albcre*^*−*/*−*^ female mice with *Prkn*^fl/fl^-*Albcre*^*−*/+^ male mice. Twelve-week old WT and LKO mice were fed a western diet (WD; kcal distribution 44.4% carbohydrate, 40.1% fat, 15.5% protein, AIN-76A Western Diet 5342, catalog #1810060, Test Diet; drinking water supplemented with 23.1 g/l fructose and 18.9 g/l glucose) for six or 20 weeks prior to study. For hyperinsulinemic euglycemic clamp studies, six-week old mice were fed WD for two weeks prior to surgery and five additional days post-surgery prior to the clamp. For mito-Keima studies, twelve-week old male and female hemizygous mice on the FVB/NJ background strain were randomly assigned to control regular chow (RC; kcal distribution 59.5% carbohydrate, 14.4% fat, 26.1% protein, ProLab IsoPro 5P76, catalog #0006972; regular drinking water) or WD. Male mice were maintained on diet for one, six, 11 and 16 weeks and females were maintained on diet for 6 and 11 weeks. Body weight and composition (fat/lean mass by ^1^H-NMR) were measured prior to and at the end of dietary interventions. Mice were sacrificed with ad libitum access to food between 7 and 10am for all studies.

### Mitophagy flux

Differences in mitophagy rates were measured using fresh liver slices from mito-Keima mice imaged with confocal microscopy as previously described^14^. The left lobe of the liver was rapidly excised during dissection and the middle portion of the left lobe was sectioned by hand with a scapel to produce a liver slice approximately one mm thick for imaging. The liver slice was placed in a recessed, glass bottom petri dish (MatTek, P35G-1.5-14-C) and overlaid with a small volume of 25 mM Tris–HCl (pH 7.4) and a glass cover slip. Imaging was initiated within five minutes of euthanasia. Imaging was conducted using a Nikon Spectral A1 confocal microscope. Mitophagy imaging was performed with excitation wavelengths set at 561 nm (acidic or lysosomal; red) and 458 nm (neutral or mitochondrial; green) and emission spectra collected over 570–695 nm. Six images per sample were collected over approximately eight to ten minutes. Images were analyzed using NIS-Elements software and the total number of red mitophagic events were divided by the sum of the green non-mitophagic events and red events to yield the relative rate of mitophagy, as previously described^14^. Data are expressed as fold-change relative to the RC group for each time point.

### Biochemical analyses

An Analox GM9 Glucose Analyzer was used to measure glucose levels via the glucose oxidase method. Plasma insulin levels were determined using a Stellux Chemiluminescent Rodent Insulin ELISA (Alpco). Plasma and liver triglycerides were measured using Infinity Triglyceride Reagent (Thermo Fisher TR22421). Liver triglycerides were extracted using a methanol/chloroform-based method^15^ and the colorimetric Infinity Triglyceride Reagent. Plasma cholesterol levels were measured using Wako Diagnostics Total Cholesterol Kit (999–02601). Plasma fatty acids were measured using Wako Diagnostics NEFA-HR kit (999–34691, 995–34791, 991–34891, 993–35191). Plasma liver enzymes were assessed using Thermo Fisher Infinity alanine aminotransferase and aspartate aminotranferase activity kits (TR71121, TR70121).

### Histology

All histological samples were prepared and stained in the Pitt Biospecimen Core at the University of Pittsburgh Department of Pathology. Excised left lobe of the liver from each mouse at the time of dissection was fixed in 4% formalin overnight, washed, and then stored in 70% ethanol prepared with phosphate-buffered saline (PBS). Subsequently, samples were embedded in paraffin for sectioning and stained with hematoxylin and eosin (H&E) for analysis. The NAFLD activity score (NAS) was obtained based on methods established by the Pathology Committee of the Non-alcoholic Steatohepatitis (NASH) Clinical Research Network^1^. NAS was determined in a blinded independent fashion by two individuals, and the scores were averaged. NAS consisted of separate category scores made in whole-value increments for steatosis (0–3), where 0 = 0–5%, 1 = 6–33%, 2 = 34–66%, and 3 = 67–100% of hepatocytes positive for steatosis; inflammation (0–3), where 0 = no foci, 1 = more than 2 foci per 200X field, 2 = 2–4 foci per 200X field and 3 = greater than 4 foci per 200X field; and ballooning (0–2), where 0 = none present, 1 = few and 2 = many/prominent. The final NAS reported was a sum of the three category scores.

### Gene expression

RNA was isolated from liver using a Quick-RNA MiniPrep Kit (Zymo Research) and then used for cDNA synthesis using a QuantiTect Reverse Transcriptase Kit (Qiagen), both according to manufacturer's instructions. Quantitative PCR was performed using the Applied Biosystems QuantStudio3 RT‐PCR System and PowerUp SYBR Green Master Mix (Thermo Fisher). Primer sequences for the following genes were designed with IDT Real Time PCR tool and were as follows: *Gapdh* (F 5′-GTGGCAGTGATGGCATGGAC; R 5′-CAGCACCAGTGGATGCAGGG), *F4/80* (F 5′-CCAGCACATCCAGCCAAG; R 5′-ACATCAGTGTTCCAGGAGACACA), *Cd68* (F 5′-TGCGGCTCCCTGTGTGT; R 5′-TCTTCCTCTGTTCCTTGGGCTAT). Primers for *Ccl2*, *Ccl4*, *Il-1β*, *Il-6*, *Col1al*, *Col3a1* were commercially predesigned and validated primers (QuantiTect Primer Assays, Qiagen) and sequences are proprietary. The efficiency of each primer set was determined from a four point standard curve and used to calculate relative expression using *Gapdh* as reference gene. Data are expressed as the fold‐change in relative expression relative to controls.

### Hyperinsulinemic euglycemic clamps

Clamp studies were performed as previously described^15^ with minor modifications. Briefly, an indwelling catheter was surgically implanted into the right jugular vein. The mice recovered five days prior to study and were fasted six hours in the morning prior to a primed/continuous infusion of 3-^3^H-glucose (Perkin Elmer; prime: 0.7 μCi/kg over 3 min; 0.05 μCi/min basal, 0.1 μCi/min clamp) to measure basal (fasted) and insulin-stimulated (clamp) rates of glucose turnover (whole-body glucose uptake and endogenous or hepatic glucose production). Insulin was given as a primed/continuous infusion (Novolin-R, Novo Nordisk; prime dose: 16 mU/kg over 3 min; continuous dose: 2.5 mU∙kg^−1^∙min^−1^). Blood collected by tail vein massage every 10 min during study was used for plasma measurements, and a variable infusion of 20% dextrose was administered to maintain euglycemia.

### Statistical analyses

Data represent the mean ± standard error of the mean and were compared by Student’s t-test or 2-way ANOVA followed by multiple comparison testing to compare the means between treatment groups at each time point when significant, and *p* < 0.05 was considered significant. False discovery rate was controlled for using the two-stage linear step-up procedure of Benjamini, Krieger and Yekutieli where Q = 0.05.

## Results

### Reduced mitophagy is an early feature of NAFLD pathogenesis

**Figure 1:**
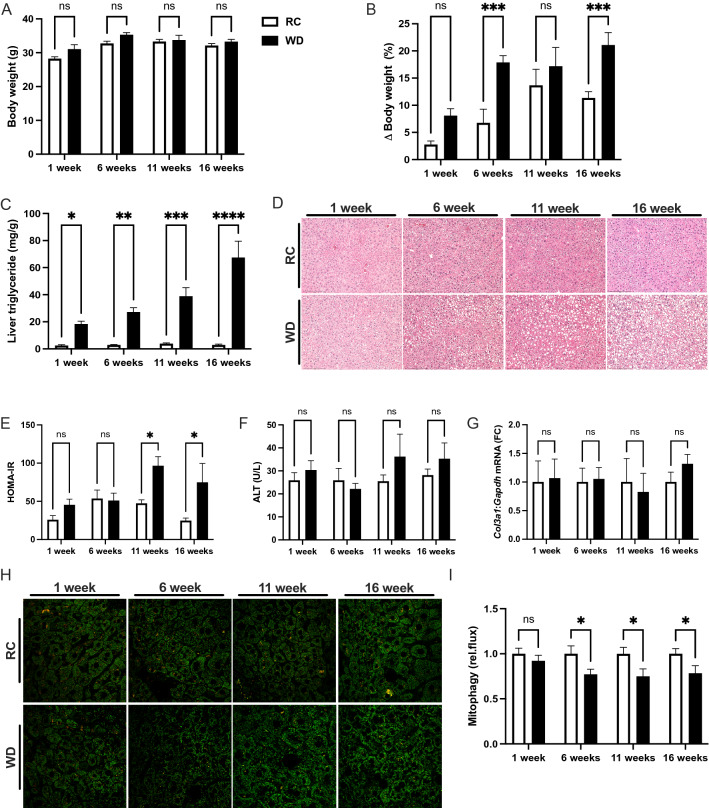
Reduced mitophagy is an early feature of NAFLD. (**A**) Body weights at one, six, 11 and 16 weeks for regular chow (RC) and western diet (WD)-fed mito-Keima mice. (**B**) Change in body weight expressed as the percent increase from baseline prior to initiating WD. (**C**) Liver triglyceride levels expressed as mg triglyceride per g tissue. (**D**) Representative H&E stained liver sections from mito-Keima mice at each experimental timepoint. (**E**) HOMA-IR calculated from plasma glucose and insulin levels as a surrogate for insulin resistance. (**F**) Plasma ALT levels from mito-Keima mice at each timepoint. (**G**) QPCR data showing gene expression of *Col3a1* relative to *Gapdh* expressed as the fold-change relative to RC-fed mice at each timepoint. (**H**) Representative images from confocal microscopy used to determine relative rates of mitophagy using mito-Keima. (**I**) Relative rates of mitophagy calculated as the ratio of red signal to the total of red and green signal, expressed as fold-change relative to RC group at each timepoint. Data are the mean ± s.e.m. for n = 5–10 mice per group. Data were analyzed by 2-way ANOVA followed by multiple comparison testing. *p < 0.05, **p < 0.01, ***p < 0.001.

Mitochondrial dysfunction is associated with NAFLD pathogenesis^27–29^. Reduced mitophagy may contribute to the observed changes in mitochondrial function in this context^12,15,30^. Reduced rates of hepatic mitophagy were previously observed in mito-Keima mice after long-term high-fat diet feeding for 18 weeks^14^. To determine when reduced rates of mitophagy occur during the pathogenesis of NAFLD, we fed mito-Keima mice RC or WD for one, six, 11 and 16 weeks. The mito-Keima mice were created on the FVBN background, a strain known to gain less weight when fed an obesogenic diet compared with more common strains, such as C57BL6^31–33^. There was a modest but significant effect of diet on body weight and a significant effect of time (*p* < 0.05, *P* < 0.001 by 2-way ANOVA) and multiple comparison testing detected no differences in body weight at any timepoints (Fig. 1A). When the change in body weight was expressed as the percent change prior to dietary intervention, diet and time, again, produced significant effects on body weight (*p* < 0.001, *p* < 0.001 by 2-way ANOVA) and differences between RC and WD mice were significant at six and 16 weeks (Fig. 1B). The differences in body weight were attributable to significant increases in fat mass due to diet and time (*p* < 0.001, *p* < 0.01 by 2-way ANOVA), where differences between RC and WD mice were significant at one, six and 16 weeks (Fig. 1SA). There was also a significant effect of diet and time on lean mass (*p* < 0.05, *p* < 0.05 by 2-way ANOVA) and the between group difference was only significant at 11 weeks (Fig. S1B). Despite the modest absolute effects of WD on body weight and adiposity, there was a marked effect of WD and time on liver triglyceride levels (*p* < 0.001, *p* < 0.001 by 2-way ANOVA). Increased liver triglyceride levels were significant after one, six, 11 and 16 weeks and ranged from 7.5- to 23-fold elevated from one to 16 weeks when comparing RC to WD mice (Fig. 1C). Histological analysis demonstrated that increases in liver triglyceride levels were due to the presence of primarily micro- as opposed to macro-steatosis (Fig. 1D). There was a significant effect of diet and time on the composite NAFLD activity score (NAS; *p* < 0.0001, *p* < 0.01 by 2-way ANOVA) and differences between RC and WD were present at each time point (Fig. S1C). Differences in NAS due to WD and time resulted primarily from increases in the steatosis score (Fig. S1D; *p* < 0.001, *p* < 0.001 by 2-way ANOVA), although there was a significant effect of diet but not time on inflammation, and a significant effect of diet and time on ballooning (*p* < 0.001, *p* = 0.82, and *p* < 0.001, *p* < 0.05, respectively, by 2-way ANOVA), despite the overall low quantitative scores that averaged less than one for inflammation and ballooning for WD-fed mice at all time points (Fig. S1E-F). Qualitative assessment of systemic insulin resistance by HOMA-IR demonstrated a significant increase due to WD but not time (*p* < 0.01, *p* = 0.1 by 2-way ANOVA) and differences between RC and WD were detected at 11 and 16 weeks (Fig. 1E). The differences in HOMA-IR were due to a significant effect of WD but not time on both plasma glucose and insulin levels (*p* < 0.01, *p* = 0.06, and *p* < 0.05, *p* = 0.13 by 2-way ANOVA, respectively; Fig. 1SG-H), although there were no between group differences for RC versus WD at any timepoints. Plasma cholesterol levels were significantly increased by WD and time and there was no effect of WD and a significant effect of time on plasma fatty acid levels, and no between group differences (Fig. S1I-J). Interestingly, despite clear evidence that WD induced the metabolic aspects of NAFLD, namely steatosis, insulin resistance and hypercholesterolemia, there was no effect of WD or time on other features of NAFLD including changes in plasma liver enzyme levels, nor expression of inflammatory or fibrotic markers in liver (Fig. 1F,G; Fig. 1SK-M). The metabolic enzyme stearoyl-CoA desaturase-1 (*Scd1*) was increased by WD but unaffected by time, consistent with previous observations in mice fed high fructose or saturated fat diets ^34^ (Fig. S1N). Consistent with the previous report demonstrating a suppressive effect of high-fat diet on relative rates of mitophagy at 18-weeks^14^, we observed that WD but not time had a significant effect on mitophagy (*p* < 0.001, *p* = 0.73 by 2-way ANOVA) and that there was a consistent ~ 25% significant reduction in mitophagy at six, 11 and 16 weeks and no difference after one week (Fig. H-I). We performed an abbreviated timecourse study in female mito-Keima mice, focusing on six and 11 weeks of WD feeding. In contrast to males, there was no effect of WD on body weight or fat mass in female mice (Fig. S2A-C), although there was a significant effect of time on both measures (*p* < 0.01, *p* < 0.01, respectively). Liver triglyceride levels were significantly increased by diet but not time (*p*,0.001, *p* = 0.15) with significant between group differences at both time points. Relative rates of mitophagy were significantly reduced by WD but not time (*p* < 0.01, *p* = 0.75 by 2-way ANOVA), where the effect was significant at 11 weeks and there was a trend towards reduced mitophagy at six weeks (Fig. S2E-F; *p* = 0.06 at six weeks). These data demonstrated that reduced rates of mitophagy are a sex-independent, early feature of NAFLD pathogenesis that occur in association with the metabolic but not inflammatory or fibrotic features of the disease.

### Liver-specific deletion of *Prkn* hastens the onset of multiple features of NAFLD

**Figure 2:**
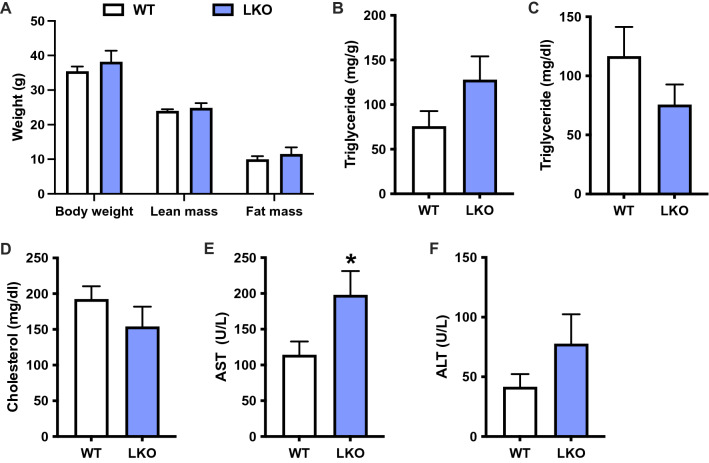
Liver injury is exacerbated after short-term western diet feeding in liver-specific PARKIN knockout mice. (**A**) Body weight and composition (fat and lean mass) for WT and LKO mice after 6 weeks WD feeding. (**B**) Liver triglyceride levels expressed as mg triglyceride per g liver. (**C**) Plasma triglyceride levels. (**D**) Plasma cholesterol levels. (**E**) Plasma AST levels. (**F**) Plasma ALT levels. Data are the mean ± s.e.m. for n = 6–7 mice per group. Data were analyzed by Student’s t-test. *p < 0.05.

PARKIN LKO mice were previously shown to be more susceptible to steatosis and hepatic insulin resistance when fed an obesogenic, 12-week high-fat that is reported to produce the metabolic but not inflammatory or fibrotic features of NAFLD^15^. To determine whether loss of mitophagy in response to a WD (Fig. 1H,I) contributed to NAFLD progression or was rather an association, we fed littermate WT and PARKIN LKO mice WD for six weeks. Six weeks of WD feeding was chosen as an early timepoint in the development of NAFLD where steatosis and insulin resistance are established, but markers of liver injury such as circulating ALT and AST, or markers of inflammation or fibrosis have yet to change^26^. There was no difference in body weight, lean mass or fat mass between WT and LKO male mice (Fig. 2A), consistent with previous observations in high-fat diet-fed LKO mice^15^. There was a 70% non-significant increase in liver triglyceride levels in LKO male mice (Fig. 2B; *p* = 0.1) and no differences in circulating levels of triglycerides or cholesterol (Fig. 2C,D). There was a significant increase in circulating AST levels in LKO compared with WT male mice, and ALT levels were approximately doubled, although the difference was not significant (Fig. 2E,F; *p* = 0.18). The effects of WD on female LKO mice were similar to male mice with the exception of liver triglyceride levels; there were no differences in body weight or composition, nor in circulating lipid levels, and circulating AST was significantly increased in LKO female mice, despite no effect of PARKIN deletion on hepatic steatosis (Fig. S3A-F).

**Figure 3:**
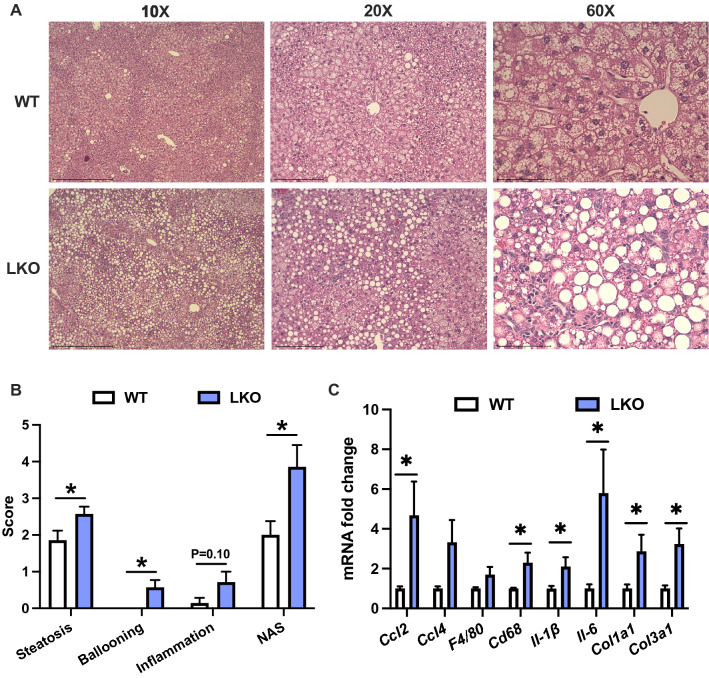
NAFLD activity score and markers of inflammation and fibrosis are increased in short-term western diet-fed liver-specific PARKIN knockout mice. (**A**) Representative images of H&E-stained liver sections from six-week WD fed WT and LKO mice at 10X, 20X and 60X. (**B**) NAFLD activity score (NAS) consisting of steatosis (0–3), inflammation (0–3) and ballooning (0–2) grading and the composite (summed criteria) NAS. (**C**) Liver gene expression measured by QPCR for noted gene markers of inflammation and fibrosis. Target gene expression was calculated relative to *Gapdh* and expressed as fold-change relative to WT. Data are the mean ± s.e.m. for n = 6–7 mice per group. Data were analyzed by Student’s t-test. *p < 0.05.

Histological examination of liver sections from WD-fed WT male mice demonstrated a typical pattern of steatosis where zones one and two appeared to contain more lipid than zone three, whereas steatosis in LKO male mice occurred in all three zones (Fig. 3A). Liver fat in both WT and LKO male mice consisted of micro- and macro-vesicular steatosis although the size and frequency of the macro-steatosis appeared greater in the LKO mice. Liver sections were scored in a blinded fashion by two individuals for NAFLD activity score and demonstrated a significantly increased composite activity score in the LKO mice. The increased overall score resulted from significant increases in the steatosis and ballooning scores, as well as a non-significant increase in the inflammation score (Fig. 3B; *p* = 0.1). Expression of several markers of immune cell recruitment, tissue immune cell content or immune cell activation were significantly increased in LKO compared with WT male mice (Fig. 3C). Specifically, *Ccl2*, *Cd68*, *Il-1β*, and *Il-6* were significantly increased (Fig. 3C). Molecular markers of fibrosis, namely *Col1a1* and *Col3a1*, were also threefold greater in LKO compared with WT male mice (Fig. 3C). In contrast, liver histology, NAS and mRNA levels of inflammatory and fibrotic markers were similar between WT and LKO female mice fed WD for six weeks (Fig. S4A-C). These data suggested that loss of PARKIN-mediated mitophagy accelerated the onset of the inflammatory and fibrotic features of NAFLD in response WD feeding in male but not female LKO mice.

### Liver-specific deletion of *Prkn* does not affect the long-term severity of NAFLD

**Figure 4:**
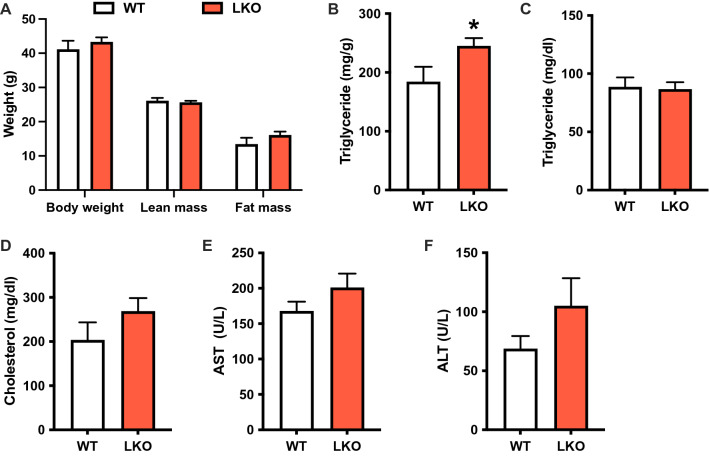
Liver steatosis but not damage is increased after long-term western diet feeding in liver-specific PARKIN knockout mice. (A) Body weight and composition (fat and lean mass) for WT and LKO mice after 20 weeks WD feeding. (**B**) Liver triglyceride levels expressed as mg triglyceride per g liver. (**C**) Plasma triglyceride levels. (**D**) Plasma cholesterol levels. (**E**) Plasma AST levels. (**F**) Plasma ALT levels. Data are the mean ± s.e.m. for n = 7–8 mice per group. Data were analyzed by Student’s t-test. *p < 0.05.

Next, we evaluated the effects of liver-specific *Prkn* deletion on NAFLD progression following 20-weeks of WD feeding. Twenty weeks was chosen as a later time point in the pathogenesis of NAFLD when the full spectrum of liver disease including steatosis, inflammation and fibrosis are present in WT mice^26^. There were no differences in body weight, lean mass or fat mass between WT and LKO male mice (Fig. 4A). Liver triglyceride levels were significantly increased by 30% in LKO mice compared with WT male mice (Fig. 4B), similar to the previous report on *Prkn* LKO mice fed high-fat diet for 12 weeks^15^. There were no differences in plasma triglyceride or cholesterol levels, and similarly, plasma AST and ALT were not different between genotypes (Fig. 4C–F). Similarly, there were no differences in body weight or composition, circulating lipids or liver enzymes, or hepatic triglyceride levels between female WT and LKO mice (Fig. S5A-F).

**Figure 5:**
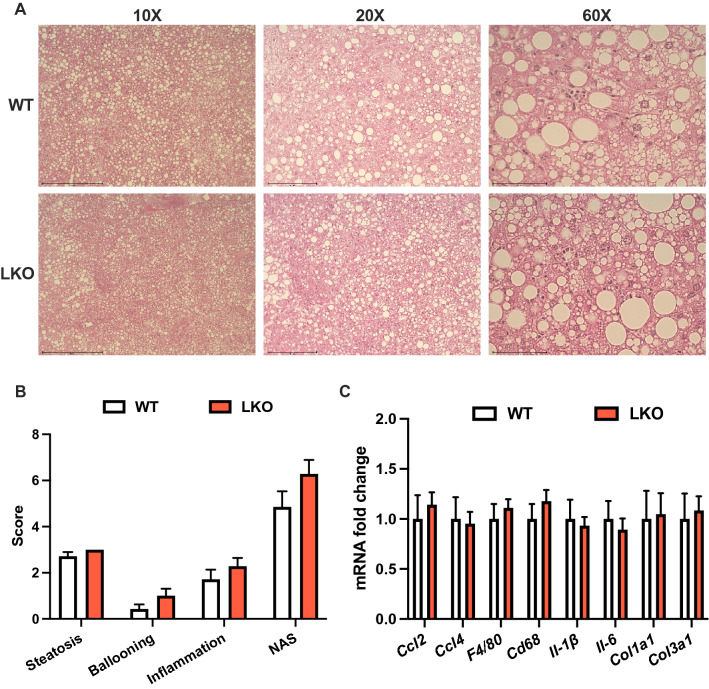
Liver-specific PARKIN knockout does not affect the NAFLD activity score or markers of inflammation and fibrosis after long-term western diet feeding. (**A**) Representative images of H&E stained liver sections from 20-week WD fed WT and LKO mice at 10X, 20X and 60X. (**B**) NAFLD activity score (NAS) consisting of steatosis (0–3), inflammation (0–3) and ballooning (0–2) grading and the composite (summed criteria) NAS. (**C**) Liver gene expression measured by QPCR for noted gene markers of inflammation and fibrosis. Target gene expression was calculated relative to *Gapdh* and expressed as fold-change relative to WT. Data are the mean ± s.e.m. for n = 7–8 mice per group. Data were analyzed by Student’s t-test.

Histological analysis of liver sections from WT and LKO male mice demonstrated striking steatosis that was evident in zones one, two and three in sections from both genotypes, as well as the presence of inflammatory cell infiltrate and ballooned hepatocytes (Fig. 5A). Steatosis consisted of both micro- and macro-vesicular steatosis and the pattern of steatosis appeared similar between groups. Scoring for NAFLD activity score features suggested advanced steatosis, mild balllooning and moderate levels of inflammation in both groups. Importantly, there was no difference in the composite NAFLD activity score nor any of the individual criteria scores between groups (Fig. 5B). Expression of the same panel of inflammatory and fibrotic markers as measured at six weeks demonstrated no differences between WT and LKO male mice after 20 weeks of WD feeding (Fig. 5C). Analysis of these same parameters in female WT and LKO mice fed WD for 20 weeks also demostrated no effect of PARKIN deletion (Fig. S6A-C). These data suggested that WT and LKO mice converged phenotypically with regards to inflammation and fibrosis as the severity of the NAFLD phenotype plateaued (see discussion for details on this point), but that there was a persistent affect to exacerbate steatosis in male mice.

### Hepatic insulin sensitivity is impaired in liver-specific *Prkn* knockout mice following short-term dietary stress

**Figure 6:**
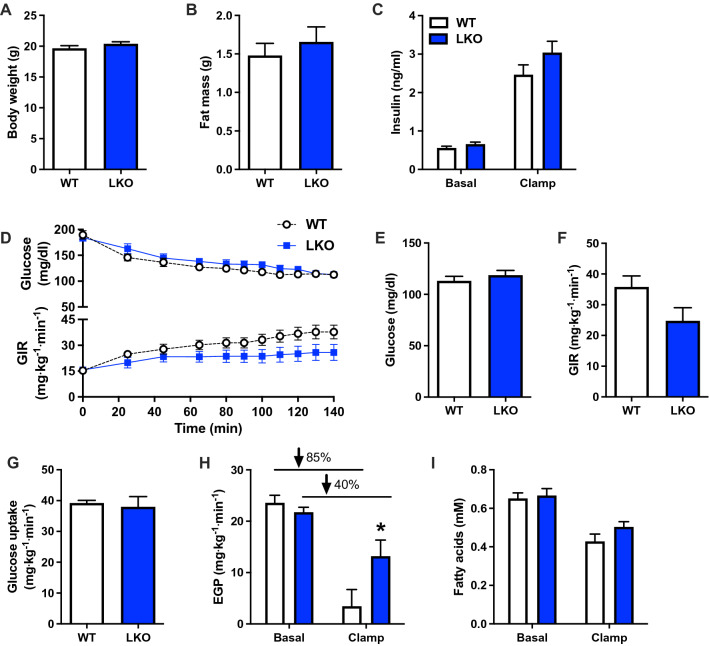
Hepatic insulin sensitivity is reduced in liver-specific PARKIN knockout mice after acute western diet feeding. (**A**) Body weights prior to hyperinsulinemic euglycemic clamp. (**B**) Fat mass prior to hyperinsulinemic euglycemic clamp. (**C**) Basal (6 h fasted) and clamped plasma insulin levels. (**D**) Plasma glucose levels (upper panel) and the glucose infusion rate (GIR; lower panels) during the clamp. (**E**) Clamped plasma glucose levels during steady-state or the final 40 min of the clamp. (**F**) GIR during steady-state. (**G**) Whole-body glucose uptake during steady-state. (**H**) Basal and clamped rates of endogenous (hepatic) glucose production (EGP). Percentages represent the percent suppression of EGP in fasted mice by insulin during the clamp. (**I**) Basal and clamped plasma fatty acid levels. Data are the mean ± s.e.m. for n = 9–10 mice per group. Data were analyzed by Student’s t-test. *p < 0.05.

We previously demonstrated that liver mitochondrial respiratory capacity was impaired in PARKIN LKO mice and that whole-body insulin sensitivity was reduced after 12-weeks of high-fat diet feeding^15^. The short- and long-term WD challenges reported above suggested that loss of PARKIN in hepatocytes accelerated the onset of NAFLD, but that over time WT and LKO mice converged phenotypically as NAFLD severity plateaud. To determine if insulin resistance in response to WD followed a similar pattern, we subjected WT and LKO mice to WD for three weeks prior to performing hyperinsulinemic euglycemic clamps. Three weeks high-fat diet feeding was previously demonstrated to induce hepatic insulin resistance^35^, such that we chose this duration to stress the liver. There was no difference in body weight or fat mass between groups following the three weeks of WD feeding (Fig. 6A,B). Plasma insulin and glucose levels were similar after a six-hour morning fast prior to the clamp (Fig. 6C,D). Plasma glucose levels were matched at approximately 115 mg/dL during the hyperinsulinemic infusion (Fig. 6D upper panel and Fig. 6E), and the glucose infusion rate (GIR) required to maintain euglycemia was 30% less in the LKO mice, although the difference was not significant (Fig. 6D lower panel; Fig. 6F *p* = 0.07). There was no difference in whole-body glucose uptake during the clamp (Fig. 6G), but insulin-stimulated rates of endogenous (hepatic) glucose production (EGP) were fourfold greater in LKO mice, demonstrating hepatic insulin resistance in LKO compared with WT mice (Fig. 6H; *p* < 0.05). Indeed, rates of EGP were suppressed from the basal state in response to hyperinsulinemia by approximately 85% in WT mice and only 40% in the LKO mice (Fig. 6H). There was no difference in fasting or clamped plasma fatty acid levels (Fig. 6I) and plasma insulin levels were matched during the hyperinsulinemic infusion (Fig. 6C).

## Discussion

Several important new observations were made during the time course studies focused on the effects of WD on liver mitophagy rates in mito-Keima mice and NAFLD progression in the liver-specific PARKIN knockout mice. We found that relative rates of mitophagy declined as early as six weeks after starting WD, establishing that reduced mitophagy flux is an early feature of NAFLD. Also, when considering potential mechanisms by which NAFLD may inhibit mitophagy, our observations suggest that inflammation and fibrosis are not likely to contribute. FVBN mice fed WD did not develop inflammation, fibrosis or increased levels of circulating liver enzymes, despite the presence of marked steatosis as early as one-week after initiating the WD. Further evidence in support of this idea comes from the initial report of reduced liver mitophagy in mito-Keima mice where a high-fat diet was used that produces the metabolic but not inflammatory or fibrotic features of NAFLD^14,25^. While FVBN mice are known to be resistant to CCL4-induced liver fibrosis and less susceptible to alcoholic fatty liver disease compared with other strains^36,37^, this is the first report of their protection against the inflammatory and fibrotic aspects of NAFLD in response to WD feeding of which we are aware. That fact that relative rates of mitophagy were intact one-week after starting the WD when steatosis was clearly present suggests liver lipid levels alone are insufficient to suppress mitophagy and that some other secondary change in response to steatosis likely contributes.

In parallel, we found that the onset of the primary pathogenic features of NAFLD, namely steatosis, hepatic insulin resistance, inflammation and fibrosis, occurred earlier in liver-specific PARKIN knockout mice in response to WD feeding. Interestingly, as the severity of NAFLD progressed the differences between WT and LKO mice became less apparent, suggesting WT mice ‘caught up’ or converged phenotypically with LKO mice. Notably, the physiological features of NAFLD do not progress linearly with time, but rather plateau around 16–24 weeks after initiating WD^26^. Comparing aspects of key NAFLD endpoints supports this idea; at 6 weeks, plasma AST was approximately 100 U/L in WT and 200 U/L in LKO mice (Fig. 2E) and by 20 weeks increaesd to approximately 160 U/L in WT mice whereas LKO levels remained close to 200 U/L (Fig. 4E); at 6 weeks, steatosis scores averaged 1.8 for WT and 2.6 for LKO mice (Fig. 2B) and by 20 weeks averaged 2.7 for WT and 3.0 for LKO mice (Fig. 5B). Additional evidence in support of this point comes from previous work in our lab where we performed hyperinsulinemic euglycemic clamps in WT and LKO mice after 12 weeks high-fat diet feeding^15^. Although caution should be applied when comparing data from these studies due to differences in the diets used (high-fat diet versus WD), we did observe that the overall difference in whole-body insulin sensitivity between WT and LKO mice was approximately threefold greater after three weeks WD compared with 12 weeks high-fat diet (GIR difference of 13.5 mg kg^−1^ min^−1^ here versus 5.0 mg kg^−1^ min^−1^ in reference^15^), suggesting that the metabolic phenotype of the LKO mice in response to nutritional stress is more apparent early in the disease progression. This may reflect declining rates of mitophagy in WT mice due to the WD feeding (Fig. 1), such that WT and LKO mice become more phenotypically similar with time. However, we cannot draw firm conclusions regarding this point due to the fact that we did not measure changes in mitophagy rates in the WT and LKO studies (see limitations for more on this point). Overall, these data suggest that PARKIN-mediated mitophagy protects against the immediate stress of WD and that preventing the loss of mitophagy that occurs with WD feeding may slow or prevent NAFLD progression.

Mitochondrial mass and function are, in part, maintained through the production of new mitochondria via mitochondrial biogenesis and the selective removal and degradation of dysfunctional mitochondria by mitophagy. Mitochondrial dysfunction likely contributes to disease progression via multiple mechanisms that are not mutually exclusive, including excessive and incomplete fatty acid oxidation and accumulation of lipotoxic intermediates, reactive oxygen species production, altered cellular calcium dynamics and NADH-reductive stress^38–40^. Several independent studies demonstrate that impairments in the mitophagy pathway lead to abberant mitochondrial function^41–43^, and genetic studies in mice where genes involved in regulating mitophagy are deleted implicate the loss of mitophagy specifically in liver with enhanced hepatic disease progression^15,30,44,45^. For example, BNIP3 is an atypical mitochondrial protein that contains a BH3 domain and plays a role in mitophagy by recruiting autophagosomes to mitochondria through direct interaction with the microtubule-associated protein 1-light chain 3 (LC3). Loss of BNIP3 results in increased hepatic lipid synthesis, reduced AMPK activity, elevated reactive oxygen species, inflammation, and features of NASH in mice livers, suggesting that reduced mitophagy leads to NAFLD^30^. Furthermore, genetic deletion of PARKIN accelerates acute and chronic alcohol-induced liver injury and steatosis, as well as acetaminophen-induced liver injury^44,45^, and, as noted earlier, liver-specific PARKIN knockout mice display impaired mitochondrial respiratory capacity and are more susceptible to diet-induced hepatic steatosis and insulin resistance^15^. Lastly, ALCAT1 is an acyl-CoA dependent lysocardiolipin acyltransferase that catalyzes the remodeling of aberrant cardiolipin in common metabolic diseases including obesity and type 2 diabetes, and whose expression is upregulated in liver of mouse models of obesity^46^. Overexpression of ALCAT1 in primary hepatocytes leads to several features of NAFLD including steatosis, impaired autophagy, and mitochondrial dysfunction, whereas deletion of ALCAT1 restores mitophagy and reduces steatosis, reinforcing a link between mitophagy and NAFLD^46^, as we observed here. Thus, our data add to a growing body of evidence suggesting that mitophagy plays an important role in liver by protecting against disease progression. Importantly, this function of mitophagy appears to be sex-dependent, as there were no differences in disease endpoints when comparing WD-fed female LKO and WT mice at either timepoint. Female mice may be less dependent on mitophagy to maintain liver mitochondrial homeostasis due to increased mitochondrial electron transport chain protein levels, enhanced coupling of mitochondrial respiration to ATP synthesis, and reduced reactive oxygen species production compared with males^47–49^, allowing a buffer against nutritional stress independent of mitophagy.

Although our data clearly demonstrates that nutritional stess in the form of WD feeding is associated with reduced liver mitophagy, the underlying mechanism for this effect remains a major outstanding question. One possibility is that reduced mitophagy results from a generalized loss of macroautophagy, which has been reported in models of obesity-associated fatty liver^50–52^. Notably, reduced macroautophagy in these reports occurred in extreme models of genetic obesity (*ob/ob* or *db/db*) or after long-term high-fat diet feeding of 16 weeks or more^50–52^. Whether macroautophagy is reduced in response to short-term dietary stress, such as six weeks where we first observed reduced mitophagy here in mito-Keima mice, remains untested to our knowledge.

Another possibility is that alterations in the formation and/or release of mitochondrial-endoplasmic reticulum (ER) contacts, referred to as mitochondrial associated membranes (MAMs), contributes to reduced mitophagy. PINK1 and PARKIN accumulate at mitochondrial-ER contact sites in response to mitophagic stimuli. Release of mitochondria from the ER following degradation of mitchondrial-ER tethers, such as mitofusin-2 (MFN2), is proposed as an initiating event in the mitophagy pathway that reduces mitochondrial-ER contacts^53,54^. Increased MAMs were reported in liver of rodent models and patients with advanced NAFLD, suggesting that impaired release of mitochondria from the ER contributes to reduced mitophagy^55,56^.

A final potential explanation for reduced mitophagy in the context of NAFLis that mitophagy signaling is impaired. Multiple forms of mitophagy exist and both PARKIN-dependent and independent mechanisms have been described^57^. In addition to PARKIN, BNIP3 and NIX are known to regulate mitophagy. BNIP3 and NIX are both thought to act upstream of PARKIN and may contribute to PARKIN’s mitochondrial accumulation following membrane depolarization^58–60^. Both proteins act as scaffolds capable of binding LC3, thus facilitating delivery of mitochondria to the autophagosome^58–60^. While PARKIN is best described in the context of mitochondrial damage induced mitophagy^61,62^, BNIP3’s role in mitophagy is best described in the context of cancer, hypoxia and in liver during fasting^30,63–65^. NIX’s role is best known in the context of reticulocyte maturation^60,66^ and NIX is not expressed in mouse liver^30^. Thus, reduced mitophagy is likely to involve alterations in PARKIN or BNIP3 signaling and not NIX. As described above, PARKIN-mediated mitophagy signaling involves the synthesis of poly-ubiquitin chains on outer mitochondrial membrane proteins by PARKIN. Poly-ubiquitination facilitates recognition of damaged mitochondria by adaptor proteins, such as OPTN, NDP52 and P62 that bind ubiquitin and mediate delivery to the autophagosome^61,67,68^. PARKIN catalyzes the synthesis of ubiquitin chains consisting of lysine six-, 11-, 48- and 63-based linkages to stimulate mitophagy^23,69^. Interestingly, pan-protein lysine acetylation of mitochondrial protein from liver increases dramatically during high-fat diet feeding and is associated with metabolic dysfunction^70,71^. Moreover, acetylation of lysine residues within ubiquitin are widely reported^72–75^ and acetylation of ubiquitin impaires poly-ubiquitin chain formation^76^. These observations raise the intriguing hypothesis that acetylation of outer mitochondrial membrane ubiquitin may interfere with PINK1-PARKIN-mitophagic signaling, which will require further testing.

There are several limitations to our studies that are worth noting. First, the mito-Keima and LKO mice are on different background strains of mice, FVBN and C57BL6J, respectively, that are known to respond differently to obesigenic and fibrotic stimuli. Specifically, FVBN mice gain less weight in response to over-nutrition compared with C57BL6 mice and are protected against CCL4-induced hepatic fibrosis, while both strains develop hepatic steatosis and insulin resistance with high-fat or WD feeding^31–33,36,37^. Thus, caution should be applied when comparing results from these two different approaches. Also, dietary durations were different between mito-Keima and LKO studies, limiting potential comparisons and the experiments are better considered separately. Second, we did not measure rates of mitophagy in WT and LKO mice. Therefore, the strongest conclusion we can draw from these studies is that the phenotypes described are Parkin-dependent and not mitophagy-dependent per se. Third, we did not measure changes in insulin sensitivity by hyperinsulinemic euglycemic clamp in WT and LKO mice at a later time point, such as 20 weeks WD feeding, such that we cannot definitively conclude that the hepatic insulin resistance phenotype is more pronounced early in the progression of NAFLD as opposed to later in LKO mice.

In summary, WD feeding reduced relative rates of mitophagy in liver early during the progression of NAFLD in a sex-independent fashion and was associated with the onset of steatosis but not inflammation or fibrosis. In addition, liver-specific deletion of PARKIN, a key mitophagy signaling protein, accelerated the onset of the primary features of NAFLD in a sex-dependent fashion, where male but not female mice were affected. These findings enhance our understanding of the role of the mitochondria and mitophagy in hepatic disease progression during nutritional stress. They also highlight the need for future studies to determine the mechanism by which WD and high-fat diet feeding reduce mitophagy in liver and to understand why female mice are less reliant on hepatic mitophagy to maintain mitochondrial homeostasis compared with male mice.

## Supplementary Information

Supplementary Information.

## Data Availability

Data and research materials presented here will be made readily available upon request to the corresponding author.
