# Supplementary material for: Reduced mitophagy is an early feature of NAFLD and liver-specific PARKIN knockout hastens the onset of steatosis, inflammation and fibrosis
Source: Sci Rep. 2023 May 10;13:7575. doi: 10.1038/s41598-023-34710-x (PMC10172344; doi:10.1038/s41598-023-34710-x)
Supplement: Supplementary file 1 — Supplementary Information. [file 41598_2023_34710_MOESM1_ESM.pdf]

Supplemental Figure 1

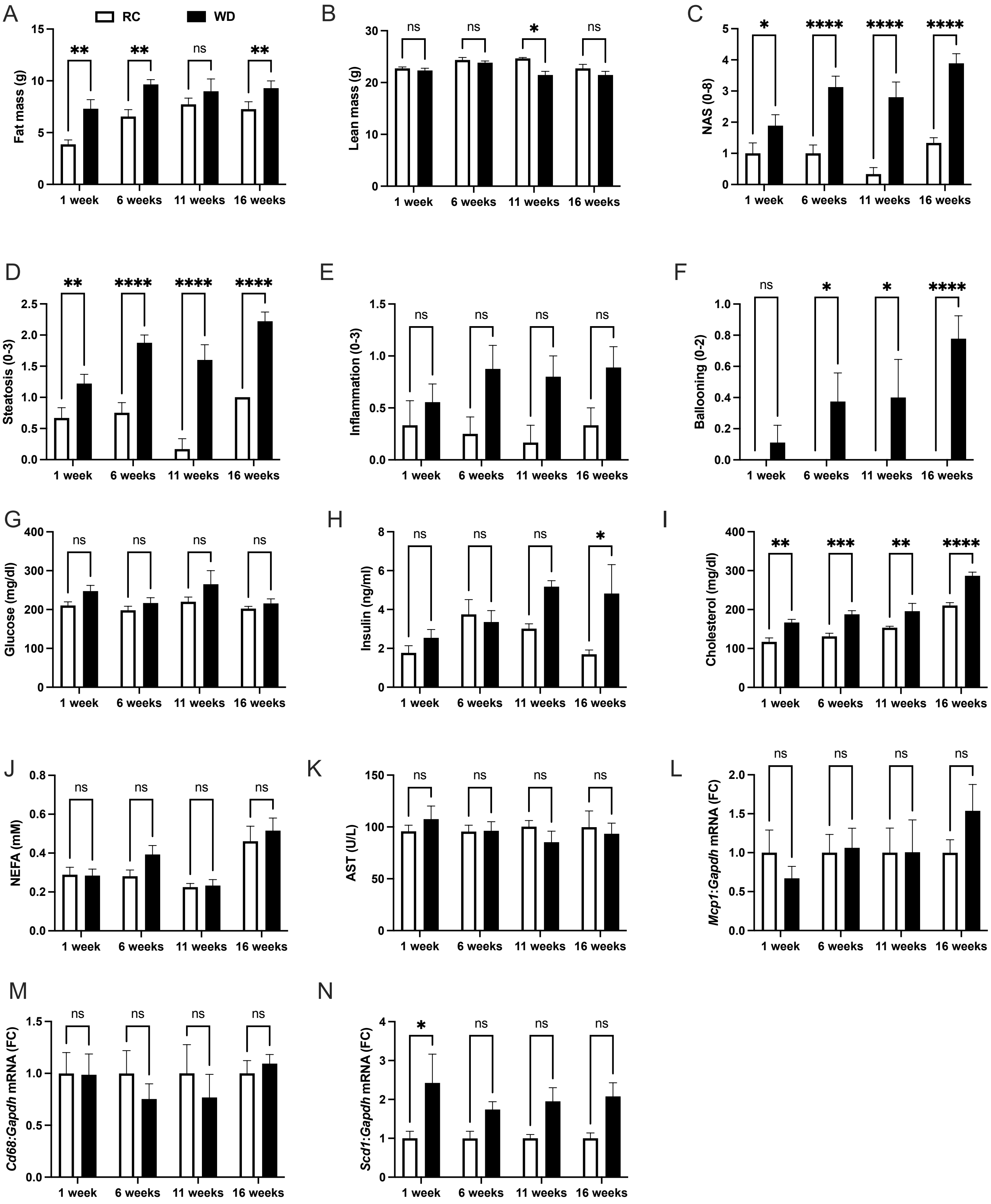

Figure S1: Characterization of male mito-Keima mice fed western diet. A-B. Fat and lean mass. **C-F.** NAFLD criteria and composite histology scores. **G-K.** Plasma glucose, insulin, cholesterol, NEFA and AST levels. **L-N.** Liver mRNA levels for *Mcp1*, *Cd68* and *Scd1*. Data are the mean ± s.e.m. for n = 5-10 mice per group. Data were analyzed by 2-way ANOVA followed by multiple comparison testing. *p<0.05, **p<0.01, ***p<0.001, ****p<0.0001.

Supplemental Figure 2

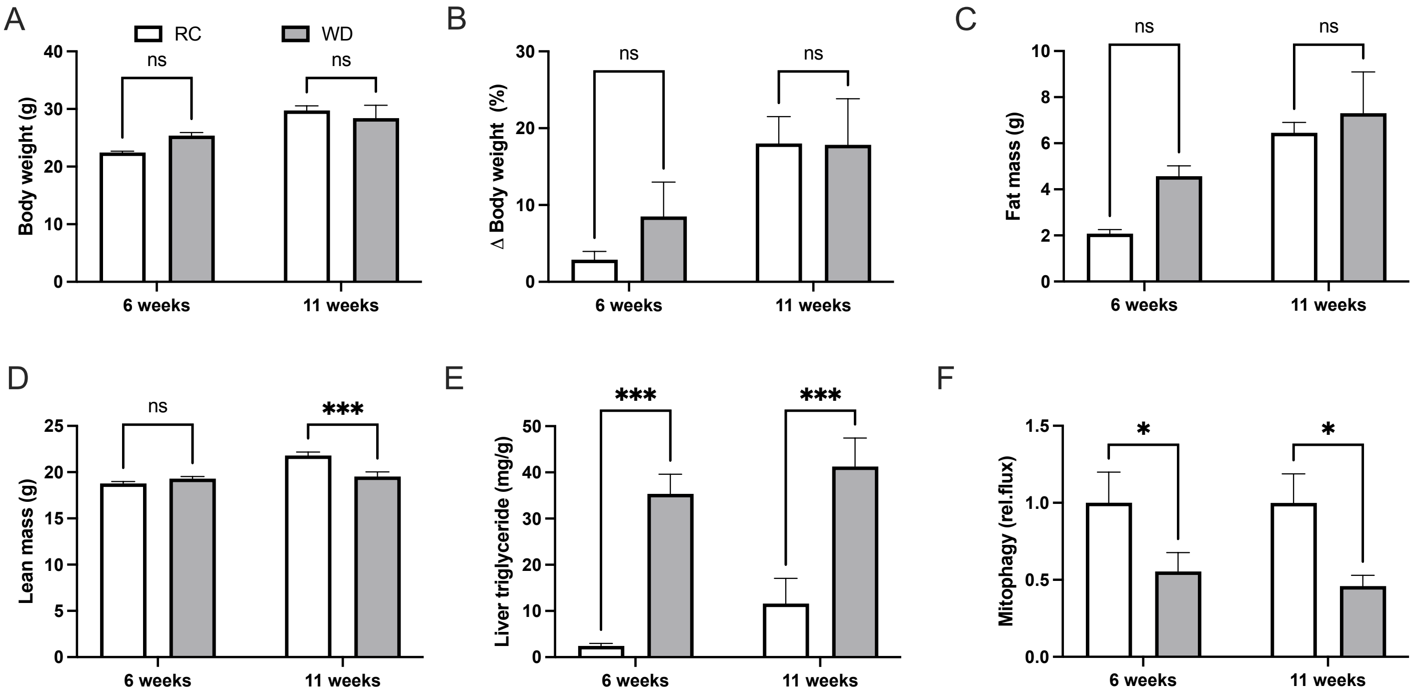

Figure S2: Characterization of female mito-Keima mice fed western diet. A-D. Body weight, change in body weight, and fat and lean mass. **E-F.** Liver triglyceride levels and relative rates of mitophagy. Data are the mean ± s.e.m. for n = 5-8 mice per group. Data were analyzed by 2-way ANOVA followed by multiple comparison testing. *p<0.05, ***p<0.001.

Supplemental Figure 3

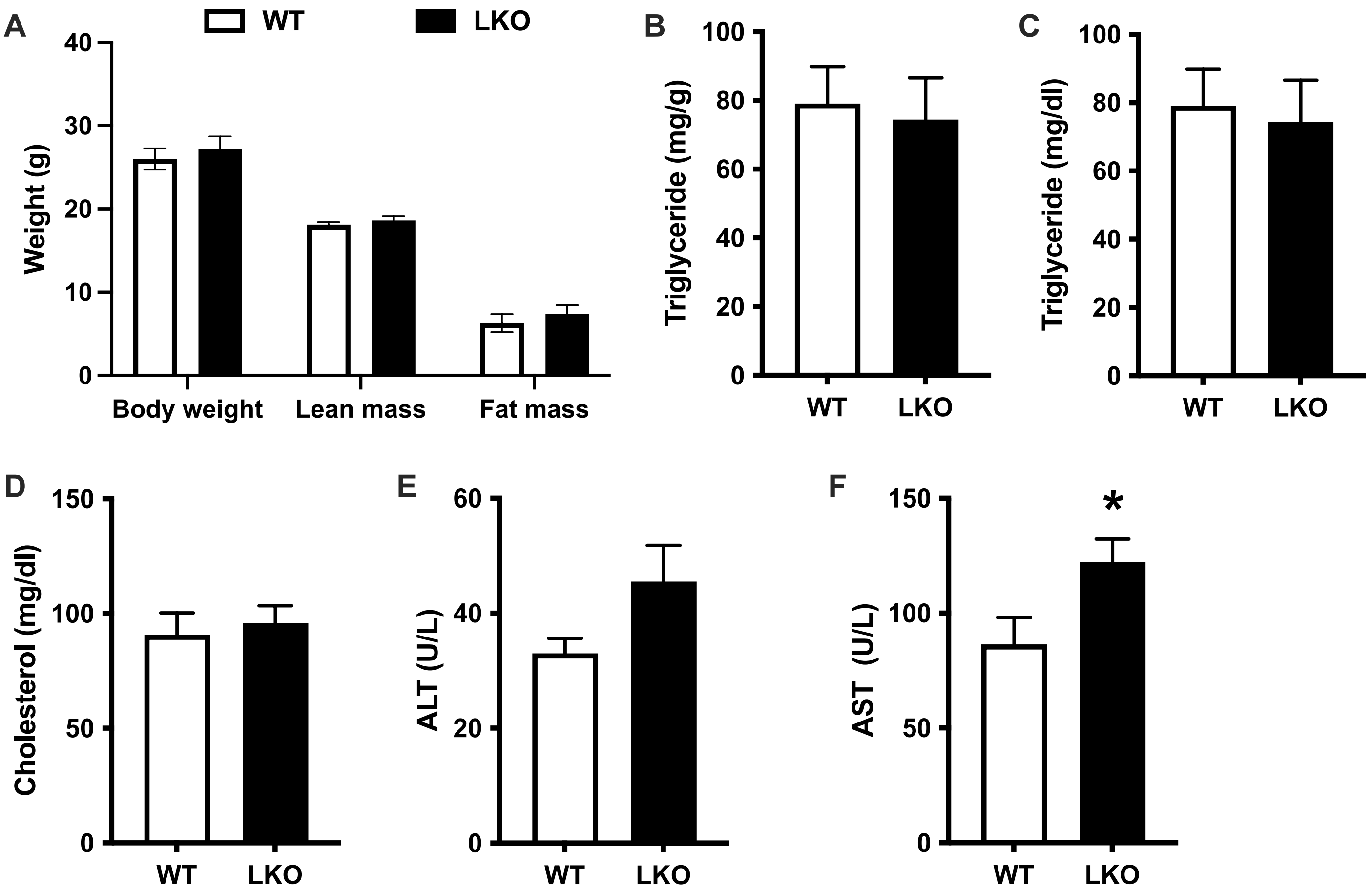

Figure S3: NAFLD progression in response to short-term dietary challange is unaffected in female liver-specific PARKIN knockout mice. A. Body weight and composition (fat and lean mass) for WT and LKO mice after six weeks WD feeding. **B.** Liver triglyceride levels expressed as mg triglyceride per g liver. **C.** Plasma triglyceride levels. **D.** Plasma cholesterol levels. **E.** Plasma ALT levels. **F.** Plasma AST levels. Data are the mean ± s.e.m. for n = 9-11 mice per group. Data were analyzed by Student's t-test. *p<0.05

Supplemental Figure 4

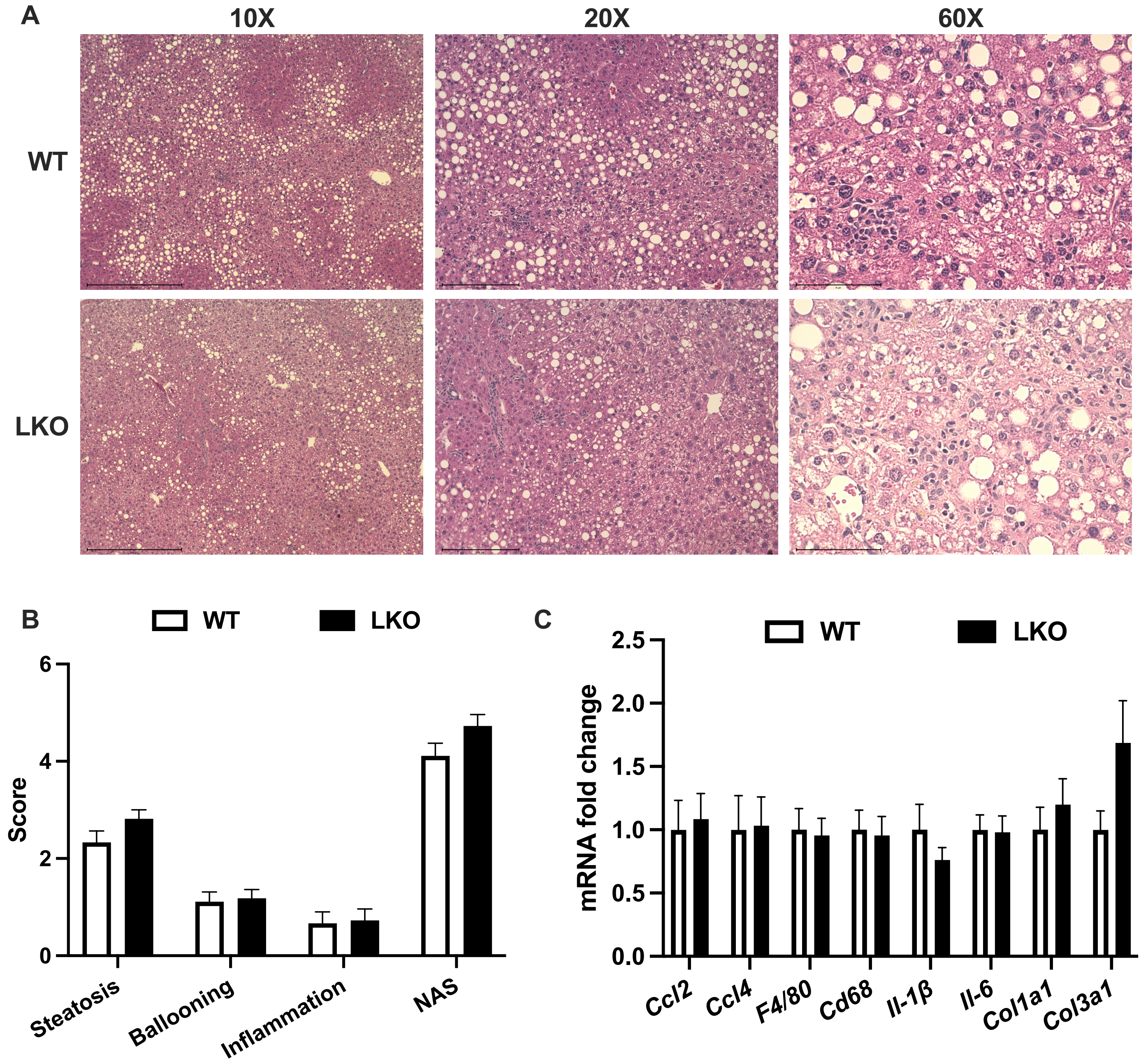

Figure S4: NAFLD progression in response to short-term dietary challange is unaffected in female liver-specific PARKIN knockout mice. A. Representative images of H&E-stained liver sections from six-week WD fed WT and LKO mice at 10X, 20X and 60X. **B.** NAFLD activity score (NAS) consisting of steatosis (0-3), inflammation (0-3) and ballooning (0-2) grading and the composite (summed criteria) NAS. **C.** Liver gene expression measured by QPCR for noted gene markers of inflammation and fibrosis. Target gene expression was calculated relative to *Gapdh* and expressed as fold-change relative to WT. Data are the mean ± s.e.m. for n = 9-11 mice per group. Data were analyzed by Student's t-test. *p<0.05

Supplemental Figure 5

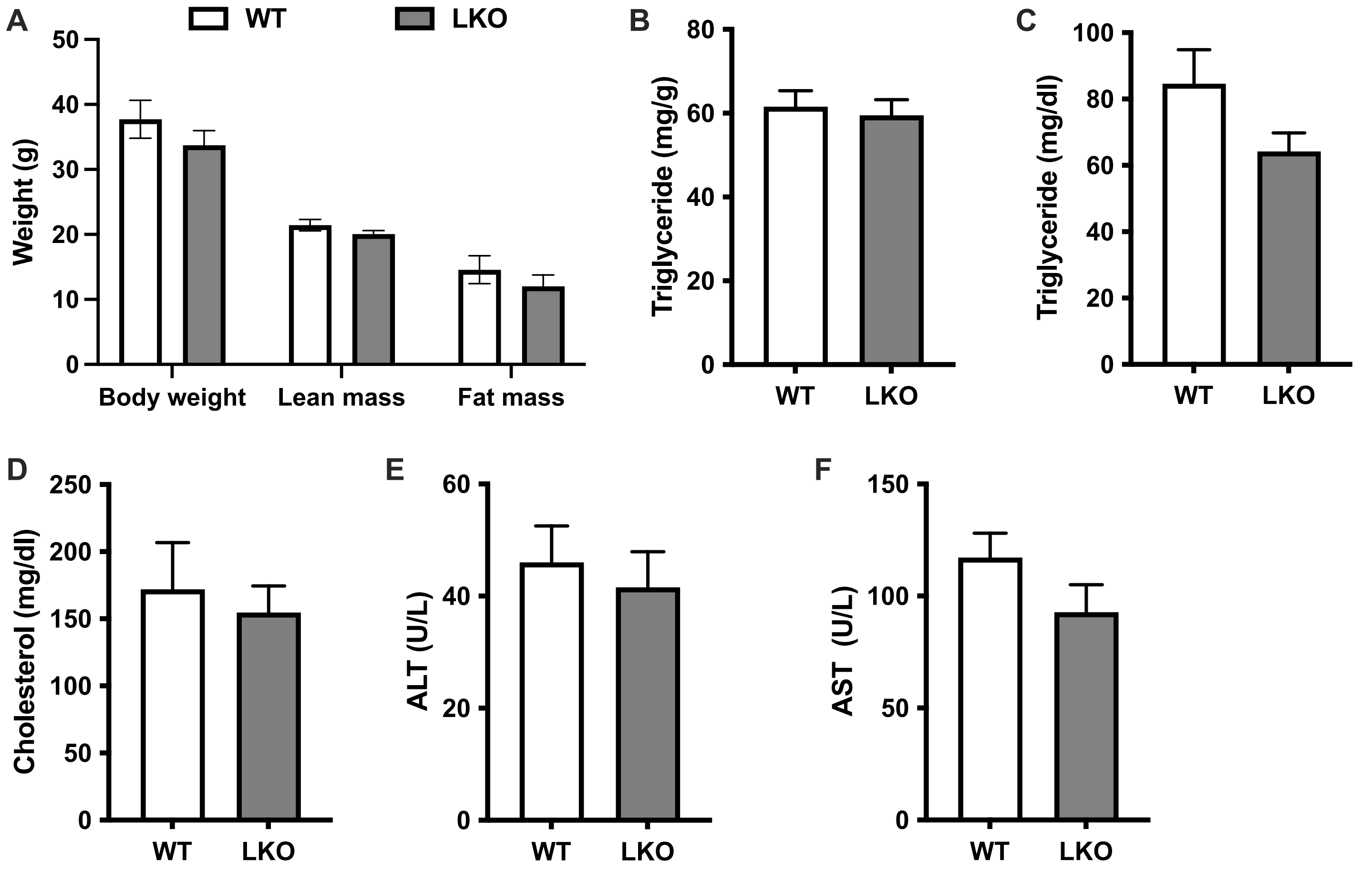

Figure S5: NAFLD progression in response to long-term dietary challange is unaffected in female liver-specific PARKIN knockout mice. A. Body weight and composition (fat and lean mass) for WT and LKO mice after 20 weeks WD feeding. **B.** Liver triglyceride levels expressed as mg triglyceride per g liver. **C.** Plasma triglyceride levels. **D.** Plasma cholesterol levels. **E.** Plasma ALT levels. **F.** Plasma AST levels. Data are the mean ± s.e.m. for n = 9-11 mice per group. Data were analyzed by Student's t-test. *$p<0.05$

Supplemental Figure 6

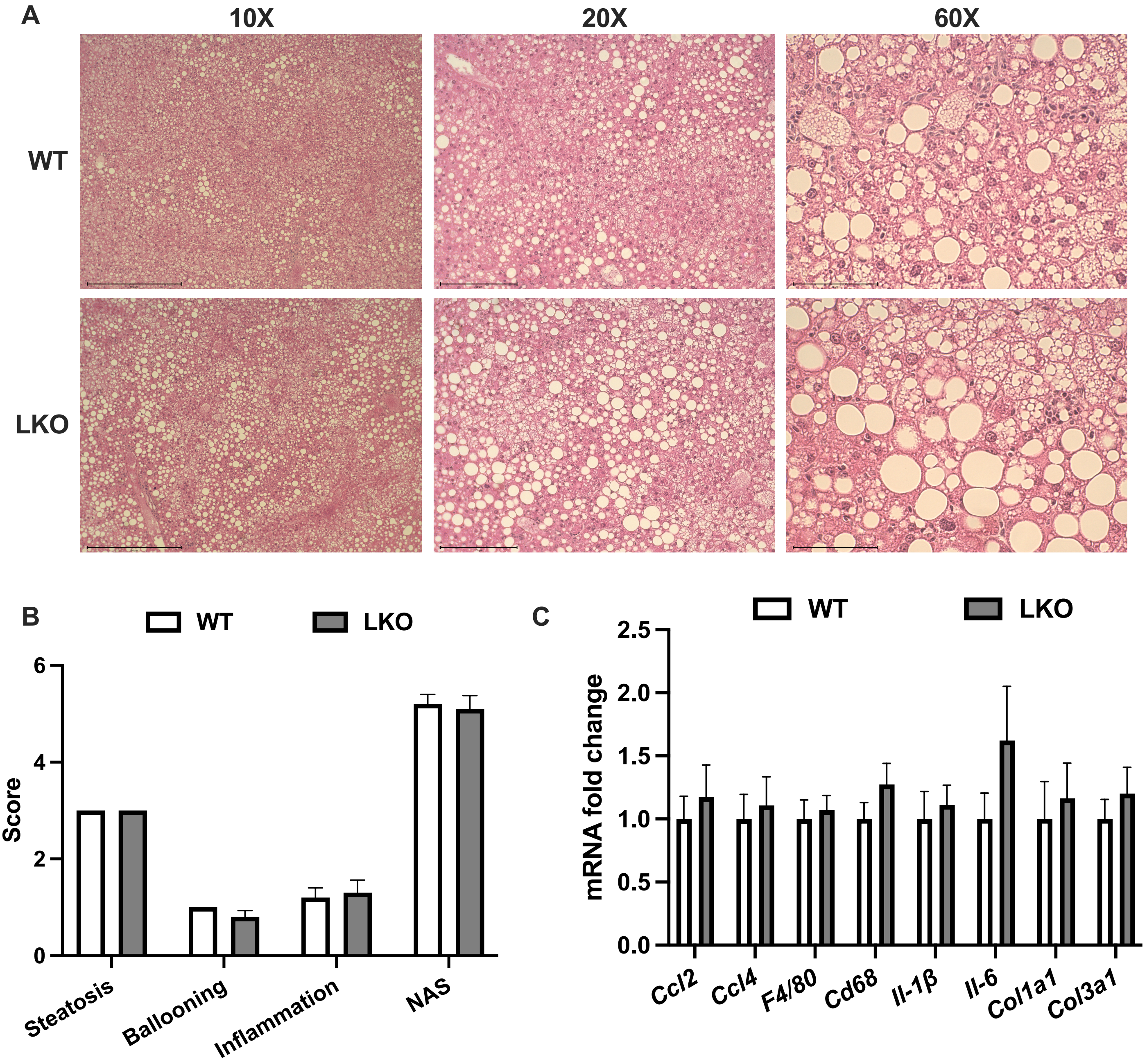

Figure S6: NAFLD progression in response to long-term dietary challange is unaffected in female liver-specific PARKIN knockout mice. A. Representative images of H&E-stained liver sections from 20-week WD fed WT and LKO mice at 10X, 20X and 60X. **B.** NAFLD activity score (NAS) consisting of steatosis (0-3), inflammation (0-3) and ballooning (0-2) grading and the composite (summed criteria) NAS. **C.** Liver gene expression measured by QPCR for noted gene markers of inflammation and fibrosis. Target gene expression was calculated relative to *Gapdh* and expressed as fold-change relative to WT. Data are the mean ± s.e.m. for n = 9-11 mice per group. Data were analyzed by Student's t-test. *p<0.05
